# Late Cutaneous Metastases of Bladder Urothelial Carcinoma: A Case Report

**DOI:** 10.7759/cureus.38038

**Published:** 2023-04-24

**Authors:** Zakariae Hayoune, Mohammed Ramdani, Youness Tahri, Ali Barki

**Affiliations:** 1 Urology, Mohammed VI University Hospital, Oujda, MAR; 2 Urology, Faculty of Medicine and Pharmacy of Oujda, Mohammed First University of Oujda, Oujda, MAR

**Keywords:** prognosis, urothelial cell carcinoma, cutaneous metastasis, skin metastases, urothelial bladder cancer

## Abstract

Cutaneous metastatic disease from bladder urothelial carcinoma is a rare but serious complication of advanced bladder cancer. It occurs when malignant cells from the primary bladder tumor spread to the skin. The most common sites for cutaneous metastases from bladder cancer are the abdomen, chest, and pelvis. We report a case of a 69-year-old patient who was diagnosed with infiltrative urothelial carcinoma of the bladder (pT2) and underwent a radical cystoprostatectomy. After one year, the patient developed two ulcerative-bourgeous lesions, which were later identified as cutaneous metastases from bladder urothelial carcinoma through histological examination. Unfortunately, the patient passed away a few weeks later.

## Introduction

The term “cutaneous metastasis” refers to the growth of a malignant tumor in the skin layer that originates in another part of the body. It may be the first sign of cancer hidden deep in the body or may indicate a sign of recurrence and/or metastatic spread of a treated tumor that was considered cured [1]. Cutaneous metastases of solid tumors are an uncommon secondary location. The overall incidence of documented skin metastasis is 2.9% of all cancer patients [2]. However, cutaneous metastases of bladder origin are extremely rare and have a dismal prognosis. Their incidence is only about 0.22% [3]. They often manifest as infiltrating plaques or nodules [4]. They may occur several months after the primary cancer or, in rare instances, as in this case, even years later.

## Case presentation

**Figure 1:**
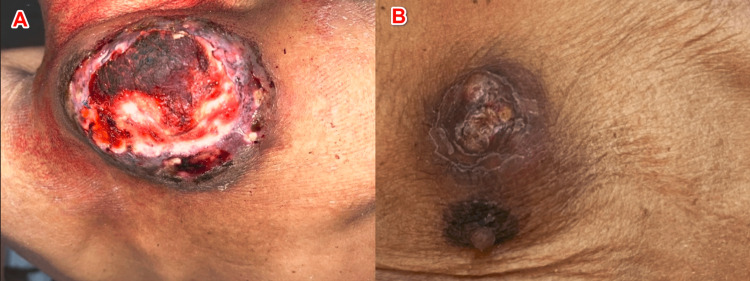
Cutaneous lesions in the posterior cervical region (A) and the right breast region (B)

Our patient is a 69-year-old male who has been a chronic smoker at a rate of 50 packs per year. He presented with total clotting hematuria for six months, accompanied by an irritative syndrome of pollakiuria and urination burn. On examination, his general condition was preserved with normocolored conjunctiva, and rectal examination revealed a fixed bladder base. Paraclinically, the complete blood count showed severe anemia at 5.1 g/dL with normal blood creatinine at 12 mg/L. The anemia had been corrected by blood transfusion, with control hemoglobin at 10 g/dL. The patient underwent a transurethral biopsy resection, which was concluded to be a muscle-infiltrating urothelial carcinoma classified as pT2. The thoracic-abdominal-pelvic computed tomography (CT) found a tumor process in the left posterolateral wall of the bladder, 61 × 37 × 34 mm in size, infiltrating the perivesical fat and the prostatic base. ﻿﻿There was no adenomegaly with pathological significance or suspicious lesion at a distance. After six courses of neoadjuvant chemotherapy with cisplatin and gemcitabine, our patient underwent a radical cystoprostatectomy with an ileal conduit, and histological examination reported a primary bladder carcinoma that was moderately to poorly differentiated grade II-III infiltrating the wall up to the serosa and the prostate without the involvement of the seminal vesicles. It was classified as pT4N0, and the tumor resection borders were healthy. The evolution was marked by the appearance of two ulcerative-bourgeous lesions of the posterior cervical region and the upper quadrants of the right breast, which appeared one year after the total cystectomy (Figure 1).

**Figure 2:**
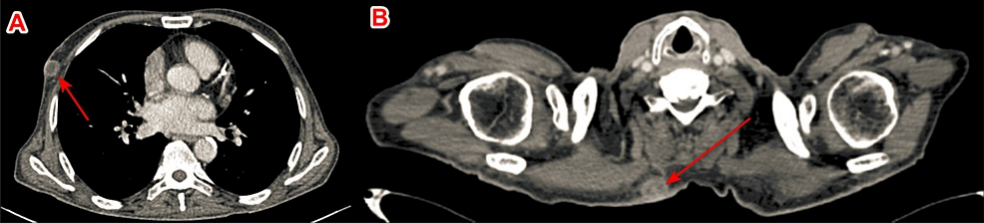
Axial section of cervical and thoracic CT scan showing cutaneous metastases in the right breast region (A) and posterior cervical region (B) CT: computed tomography

**Figure 3:**
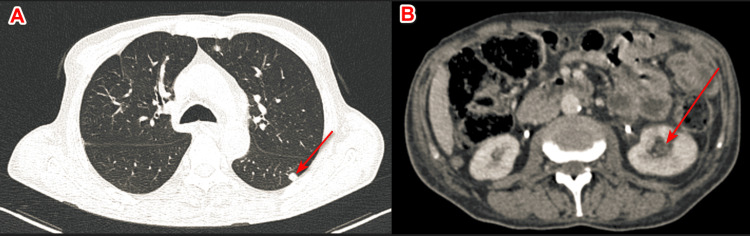
Axial section of abdominal and pelvic CT scan showing distant metastases of bladder tumor: sub-pleural and left lung parenchymal micronodule (A) and hypodense inferior polar left renal lesion with poor limitation (B) CT: computed tomography

A skin biopsy was performed, which showed a poorly differentiated carcinomatous process with focal glandular differentiation and partially necrotic. A complementary immunohistochemical study was needed, which revealed positivity for cytokeratin 7 (CK7) and GATA binding protein 3 (GATA3) compatible with a urothelial origin. An extension workup, consisting of a neck, chest, abdomen, and pelvis CT scan, was performed and revealed cutaneous, pulmonary, and renal metastases (Figures 2, 3). Palliative care was initiated, but unfortunately, the patient died a few weeks later.

## Discussion

The skin is a relatively uncommon site for metastatic dissemination of deep-seated tumors [1], accounting for only the 12th place among metastatic sites in known neoplasia [5]. The highest rates of skin metastases are observed in breast carcinoma (2.42%), followed by lung (1.78%), oral mucosa (1.75%), colon and rectum (0.81%), stomach (0.80%), and esophagus (0.74%). However, the incidence of skin metastases from vesical tumors is only 0.22%, as reported by a Taiwanese medical center [3], and they usually appear after an average delay of 18 months [6]. Lymph nodes, bones, liver, and lungs are the primary sites of metastatic spread in cases of bladder cancer [1]. Bladder cancer rarely affects the skin through various mechanisms, such as direct invasion of the tumor, spread through the blood or lymphatic system, or seeding due to iatrogenic implantation [2].

Cutaneous metastases can present with various nonspecific clinical aspects and can resemble primary skin tumors or inflammatory lesions. This similarity often leads to a delay in the diagnosis of the underlying tumor. Brownstein et al. [7] identified three main clinical presentations: nodular lesions (as in the case of our patient), sclerotic lesions, and inflammatory lesions. Therefore, performing a biopsy of any skin lesion in a cancer patient is crucial [8]. However, determining the urothelial origin can be challenging, hence the importance of immunohistochemical studies. Most of these cells express cytokeratins 7 and 20 on immunohistochemical analysis. Studies have shown that uroplakin III can be identified in approximately 50%-60% of primary urothelial carcinomas and metastatic carcinomas of urothelial origin [9,10].

The treatment of cutaneous metastases of urothelial origin is similar to that of other metastatic forms of bladder malignancy. Palliative chemotherapy is the treatment of choice. Cisplatin-based combinations, including cisplatin/gemcitabine or methotrexate/vinblastine/doxorubicin/cisplatin, are the standard first-line treatment for locally advanced unresectable or metastatic bladder tumors in patients with good general health (Eastern Cooperative Oncology Group (ECOG) performance status below 2) [11]. Patients who are ineligible for cisplatin therapy due to medical frailty or comorbidities may receive carboplatin-based regimens, such as carboplatin plus gemcitabine or carboplatin, gemcitabine, and paclitaxel, or non-platinum-based combinations, such as paclitaxel plus gemcitabine, or even monotherapy, such as gemcitabine [12]. For cisplatin-ineligible patients with high programmed death-ligand 1 (PD-L1) expression, pembrolizumab and atezolizumab are options as first-line therapy [13]. However, patients in this group have limited treatment options, and second-line therapies have modest activity with no significant improvement in patient outcomes [11]. For patients with an ECOG performance status greater than 2, palliative care is the only treatment option. Surgery is indicated for small localized lesions, while multiple lesions grouped in one area may benefit from radiotherapy [1].

Due to our patient’s deteriorated general condition and the fatal course of his disease, he was not eligible for palliative chemotherapy.

## Conclusions

Cutaneous metastasis of bladder carcinoma is a rare phenomenon that typically occurs in the later stages of the disease and is associated with a poor prognosis. Although uncommon, its diagnosis should always be considered in patients with a history of cancer. Therefore, all healthcare practitioners, including oncologists and urologists, must remain vigilant for this rare clinical presentation and inform patients accordingly to allow for early diagnosis and offer the best chances of treatment.
